# A Report of Ulcerative Colitis With Relapse on the Rectal Side of the Loop Sigmoid Colostomy and Not on the Oral Side

**DOI:** 10.7759/cureus.57941

**Published:** 2024-04-09

**Authors:** Yosuke Shimodaira, Sho Fukuda, Ryo Okubo, Kengo Onochi, Katsunori Iijima

**Affiliations:** 1 Department of Gastroenterology and Neurology, Akita University Graduate School of Medicine, Akita City, JPN; 2 Division of Gastroenterology, Omagari Kosei Medical Center, Daisen City, JPN

**Keywords:** firmicutes, inflammation, microbial composition, loop sigmoid colostomy, ulcerative colitis (uc)

## Abstract

A patient who received a loop sigmoid colostomy was diagnosed with ulcerative colitis (pancolitis type) and treated with infliximab. Thereafter, he relapsed with intestinal inflammation only on the rectal side of the loop sigmoid colostomy and not on the oral side. Autologous fecal microbiota transplantation from the proximal intestine to the distal intestine was performed to treat the inflammation but was ineffective. He was treated with oral prednisolone and induced into remission. After analyzing fecal samples from the patient, we observed an alteration of the composition of the intestinal microbiota with intestinal inflammation, including a reduction of phylum Firmicutes in the inflamed distal intestine, whereas Firmicutes was conserved in the proximal non-inflamed intestine and recovered in the distal intestine after induction of remission. Thus, our results indicated that the inflammation was associated with an alteration of the intestinal microbiota.

## Introduction

The pathogenesis of ulcerative colitis (UC) is unclear though genetic predisposition, unbalanced intestinal microbiota, and uncontrolled immune response in colonic mucosa have been associated with the disease. Although inflammation related to UC has been shown to extend proximally from the rectum, the cause of this phenomenon is not known. Studies have shown an association between UC and dysbiosis such as a decrease in the diversity of intestinal microbiota and phylum Firmicutes [1]. Alterations in gut microbiota have been noted even during inflammation and remission in UC patients, but it is unclear whether these alterations are the cause or the result of UC.

In this paper, we report a rare case of UC with relapse only on the rectal side of the loop sigmoid colostomy and not on the oral side. This study aimed to research whether microbial composition was associated with intestinal inflammation. The separated intestinal space and different inflammatory conditions in this patient, which was a rare state in UC, could benefit from this investigation. We therefore analyzed intestinal bacteria relevant to intestinal inflammation. This manuscript and the study protocol, including the autologous fecal microbiota transplantation procedure, were approved by the Akita University Graduate School of Medicine's Ethics Committee, and informed consent was obtained from the patient.

We presented this case and study in July 2023 at a medical conference of The Japanese Society of Gastroenterology.

## Case presentation

History and diagnosis

A 41-year-old man with no medical or familial history visited a clinic, complaining of rectal bleeding. A total colonoscopy revealed mild proctitis in the rectum, at which point, he was diagnosed with nonspecific colitis without abnormalities in laboratory tests. As anal swelling and pain developed, he underwent incision and drainage of the perianal abscess. One month later, the perianal subcutaneous abscess relapsed, for which he was referred to our hospital. Surgical debridement was performed on the buttocks near the anus, and the lesion was diagnosed by a dermatologist as pyoderma gangrenosum. As the anal sphincter was not functioning, loop sigmoid colostomy was performed. One month later, a colonoscopy showed fragile and edematous mucosa in the colon up to the cecum, as well as severe mixed inflammatory cell infiltration, erosion, and crypt abscess with no signs of vasculitis or Crohn’s disease on histopathological examination. Esophagogastroduodenoscopy, computed tomography, and barium radiography showed no signs of gastric or small intestinal mucosal inflammation. Stool examination showed no pathogenic bacteria and laboratory tests showed no current infectious disease of tuberculosis, Epstein-Barr virus (EBV), Cytomegalovirus, human immunodeficiency virus (HIV), hepatitis B virus (HBV), and hepatitis C virus (HCV). The patient was diagnosed with pancolitis-type UC.

Treatment

**Figure 1:**
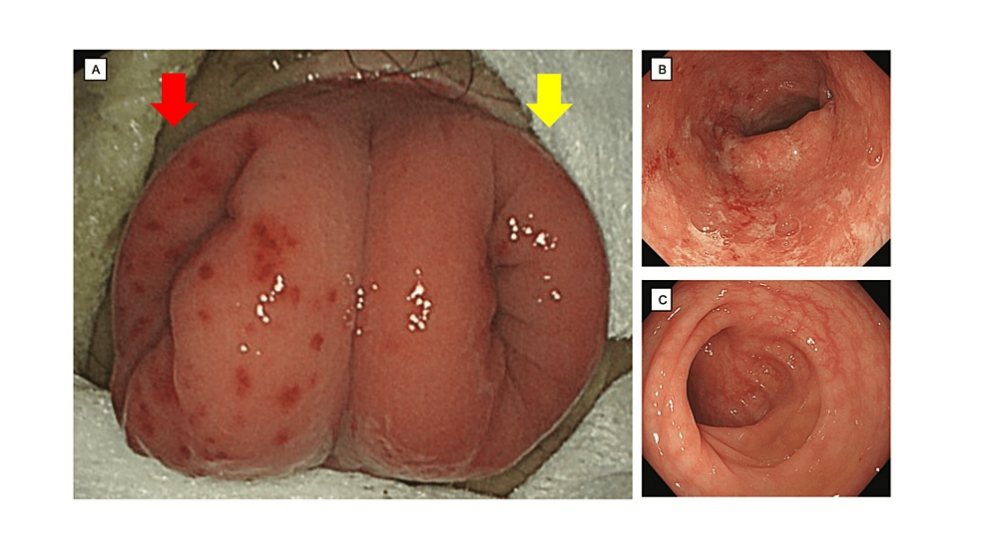
Appearance of the stoma 1A: Stoma with double orifice showing scattered erythematous spots at the end of the distal colon (red arrow) and normal mucosa at the end of the proximal colon (yellow arrow). Endoscopy revealed the following: 1B. loss of vascular marking and edematous and granular mucosa in the distal colon; 1C. mucosal healing in the proximal colon.

The patient was started on infliximab. His symptoms of abdominal pain and bloody diarrhea in the artificial anus were alleviated. Clinical remission was maintained for 12 months; thereafter, rectal bleeding occurred in the natural anus. Endoscopy revealed that the stoma leading to the natural anus showed scattered erythematous spots (Figure 1A: red arrow) loss of vascular marking, edematous and granular mucosa in the distal colon (Figure 1B), and mucosal healing on the proximal colon through its stoma (Figure 1A: yellow arrow; Figure 1C).

Attempts at fecal microbiota transplantation

Because the inflammation was clearly stopped on the anal side of the stoma, we speculated that the intestinal contents greatly contributed to the inflammation in this patient and performed an autologous fecal microbiota transplantation (FMT). The anal intestine was thoroughly washed with water to remove the intestinal contents as much as possible. Feces were obtained from the oral intestine under CO_2_ supply by endoscopy, and saline-diluted feces were immediately injected into the anal intestine. Although the procedure was repeated three times within one month, the inflammation did not improve. Finally, the patient was orally administered prednisolone, and remission was induced. He was followed for two years with infliximab and kept in remission.

Analyzing intestinal microbiota

**Figure 2:**
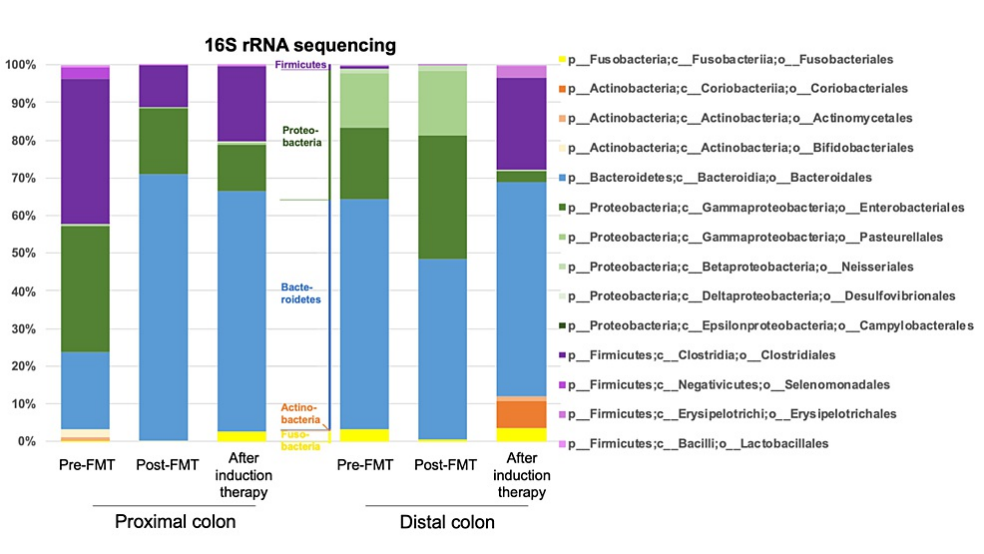
Bacterial composition of intestinal contents Intestinal contents were sampled in the proximal and distal colons. DNA was extracted with a QIAamp Fast DNA Stool Mini Kit (Qiagen, Hilden, Germany), and next-generation sequencing was performed using a MiSeq system (Illumina, Inc., San Diego, California, United States). The chart displays the phylum, class, and order of the microbiota analyzed with 16S rRNA using the QIIME2 platform. Colors represent different microbial taxa, with the legend specifying the key components.

We carefully collected feces from the proximal and distal colon through endoscopy to prevent the different sections from contaminating each other. Feces were collected at three points: immediately before the first transplantation procedure, two weeks after the third transplantation, and three months after clinical remission was introduced. The bacterial composition determined with a 16S rRNA sequencing analysis revealed that phylum Firmicutes, mostly composed of class Clostridia, was significantly less in the intestinal content of the distal colon compared to that in the proximal colon at pre-FMT. Similar Firmicutes reduction was observed in samples at post-FMT when mucosa was still inflamed in the distal colon. In contrast, an adequate proportion of phylum Firmicutes was recovered in the distal colon after induction therapy at levels similar to those in the proximal colon. Order Pasteurellales in phylum Proteobacteria, which was limited in the uninflamed colon, was increased in the inflamed distal colon (Figure 2).

Thus, our investigation of this rare case of UC featured a bacterial composition with decreased levels of phylum Firmicutes as a result of inflammation.

## Discussion

It is uncommon to perform a loop sigmoid colostomy in UC patients. Furthermore, disconnected inflammation at the stoma is rare in UC. Typically, inflammation occurs in the rectum and extends proximally in UC [2]. In this case, the disconnected inflammation developed in separate intestinal segments in one individual, which indicated that continuous intestinal access to luminal contents (e.g., intestinal microbiota and metabolites) may be a determining factor for inflammatory extension.

We performed the autologous FMT at the same time as sampling and injecting with feces, but its effectiveness was not confirmed. One of the causes of the ineffectiveness of FMT might be that the donor also had UC, with potentially “dysbiotic” microbiota even in mucosal healing. Another cause might be protocol selection for FMT, which has not yet been confirmed for UC. Some reports have shown that FMT is effective for UC, whereas others have shown the opposite [3]. The specific methods of FMT for UC (e.g., administration of antibiotics, pretreatment, and storage of stool) vary across different studies. Bacterial components, organisms other than bacteria, and metabolites could also contribute to improving UC [4]. Clinical trials, including methods optimization, are still required to determine the effectiveness of FMT for treating UC.

In this case, clinical remission and mucosal healing in the distal intestine were induced by oral prednisolone, indicating that the inflammation was a flare-up of UC only in the anal side of the intestine. We collected feces endoscopically and stored them immediately at −80 °C. The bacterial compositions determined with 16S rRNA sequencing revealed alterations of the microbiota composition, with a decrease in phylum Firmicutes consistently confirmed in the inflamed intestine, whereas it was stably conserved in the non-inflamed intestine. Of note, phylum Firmicutes was recovered after induced remission of the intestinal inflammation, suggesting that the inflammation was associated with such an alteration of bacteria. A similar report that inflamed and non-inflamed mucosa biopsied from the same patients showed different microbiota compositions supports our findings [5]. As the distal and proximal parts of the colon after colostomy are separate spaces, their microbiota could be fundamentally different. However, in this case, the alteration in the intestinal bacteria of the distal intestinal tract after the improvement of inflammation with prednisolone strongly suggested the influence of inflammation on intestinal bacteria.

Phylum Firmicutes is one of the predominant bacteria in the human intestinal microbiota [6]. Reduced Firmicutes levels have been consistently reported in UC patients. Our analysis revealed a significant reduction of Firmicutes, which was repeatedly observed in inflamed intestinal contents. Alterations in gut microbiota have also been repeatedly noted in UC patients, but it is unclear whether these alterations are the cause or the result of UC. One of the reasons for this issue may be inter-individual and intra-individual (i.e., time phase) differences in intestinal microbial communities. In this case, we analyzed the microbial composition in inflamed and uninflamed intestinal mucosa in one individual with UC at the same time.

## Conclusions

Our data indicated that mucosal inflammation was associated with microbial composition, including a reduction of phylum Firmicutes. Direct infusion with autologous FMT did not significantly modify the intestinal bacteria. Intestinal bacterial composition was shown to be affected by intestinal mucosal inflammation. Therefore, this case is a valuable addition to the literature, as it confirms alterations in intestinal bacteria as a result of inflammation.
